# Antisynthetase Syndrome in a Patient with Pulmonary Embolism and Nonbacterial Thrombotic Endocarditis

**DOI:** 10.1155/2023/9068597

**Published:** 2023-01-31

**Authors:** Anusha Vege, Jesse Beery, Areeba Kara

**Affiliations:** Department of Internal Medicine, Indiana University School of Medicine, Indianapolis, Indiana, USA

## Abstract

Antisynthetase syndrome is a rare autoimmune disease within the subset of idiopathic inflammatory myopathies. The diagnostic criteria include the presence of an aminoacyl-tRNA synthetase antibody, and typical clinical findings, including myositis, mechanic's hands, Raynaud phenomenon, unexplained fever, and interstitial lung disease. We describe a case of a 59-year-old male who presented with a 1-month history of progressive purplish discoloration and pain of the fingertips, dyspnea, cough, weight loss, fatigue, and who developed progressive proximal muscle weakness and dysphagia. Investigations revealed pulmonic valve and mitral valve marantic endocarditis, pulmonary embolism, myositis, organizing pneumonia, and elevation of anti-OJ antibodies. He was diagnosed with antisynthetase syndrome and treated with high dose corticosteroids and mycophenolate mofetil with a fair response.

## 1. Case Report

A 59-year-old man with no known medical history presented with a 1-week history of progressive and painful violaceous discoloration of his fingertips. He was a lifelong nonsmoker. He was initially diagnosed with Raynaud's phenomenon at a local emergency department (ED). However, symptoms persisted and were associated with increasing pain, bluish discoloration of the fingertips, and nonproductive cough, prompting a return to the local hospital within a few days and admission for further evaluation. Initial CT angiography of the chest showed concern for septic emboli. Transesophageal echocardiography was done due to concerns about endocarditis, which showed mitral and pulmonic valve vegetations. Blood cultures from two sets were negative without any bacterial growth. A vascular surgeon and infectious disease specialist recommended nonsurgical treatment with an empiric ceftriaxone course for 2 weeks for presumed nonbacterial thrombotic endocarditis (marantic endocarditis), and he was discharged home. Within 2 weeks, he returned with persistent ischemic fingertips and new progressive shortness of breath with minimal cough. A repeat CT angiography of the chest revealed a pulmonary embolism, and he was started on iv heparin. He was transferred to our institution for further evaluation.

On initial examination upon arrival to our hospital, he was well nourished, alert, and oriented and in no distress. He was afebrile, normotensive, with a heart rate of 100 beats per minute, a respiratory rate of 20, and an oxygen saturation of 95% while receiving 2 liters of oxygen via nasal cannula. There was no lymphadenopathy. The heart tones were regular without murmur. The lungs revealed crackles at the right basilar and left midlung fields. The abdominal exam was benign. Examination of the extremities showed ischemic changes at all fingertips. Initially, strength was normal in all extremities.

During a seven-week hospitalization, he developed worsening hypoxic respiratory failure, requiring noninvasive ventilatory support. Rapidly progressive weakness developed, with mild deltoid weakness noted during the first week of hospitalization, escalating to the need for maximal assistance for mobility and oropharyngeal dysphagia necessitating feeding tube placement by the third week.

## 2. Assessment

**Figure 1:**
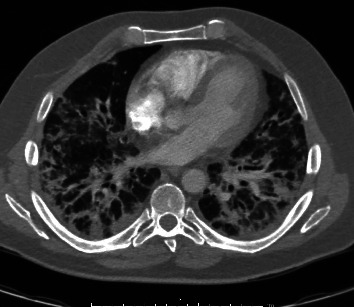
Chest CT showing bilateral airspace opacities and ground glass opacities.

A CTA of the chest with intravenous contrast showed right-sided pulmonary emboli and pulmonary infarctions and patchy bilateral airspace opacities and ground glass opacities (Figure 1). A transesophageal echocardiogram showed possible pulmonic valve vegetation and definite mitral valve vegetation measuring 8 mm × 4 mm. Blood cultures and HIV testing were negative. Bronchoalveolar lavage (BAL) cultures were negative for bacteria and fungi, and transbronchial biopsy was consistent with organizing pneumonia without evidence of pathogens.

Thrombophilia workup, including testing for factor V Leiden and prothrombin gene mutations, was negative. The lupus anticoagulant was not detected. Cardiolipin antibodies and beta-2 glycoprotein antibodies were normal.

Rheumatological testing, including ACE, C3, and C4 levels, ANA, antidouble-stranded DNA, the ANCA panel, the ENA panel, RF, anti-CCP antibodies, and cryoglobulin assays, were normal. The viral hepatitis panel was negative. Serum protein electrophoresis was negative for monoclonal protein.

**Figure 2:**
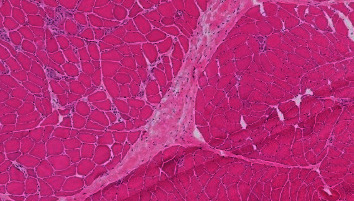
Right biceps muscle biopsy with hematoxylin and eosin staining showing necrotizing myopathy with muscle fiber necrosis and regeneration without inflammation.

Creatine kinase was elevated to 6284 units/L; aldolase was elevated to 82 units/L. An MRI of the right leg showed T2 hyperintense signal in the quadriceps, adductor, semimembranosus, and semitendinosus muscles with enhancement after administration of contrast, consistent with myositis. Electromyography of the left arm showed evidence of a myopathic process with fiber splitting, myonecrosis, and membrane instability. A right biceps biopsy showed necrotizing myopathy without inflammation (Figure 2). An extended myositis panel was done by standard immunoprecipitation, and qualitative immunoblot methodology was positive for anti-OJ isoleucyl-tRNA synthetase antibody. A diagnosis of antisynthetase syndrome was made.

## 3. Diagnosis

Initial infectious, rheumatologic, and hematologic studies did not provide a unifying diagnosis, and progressive muscle weakness prompted further evaluation. Based on the positive anti-OJ isoleucyl-tRNA synthetase antibody, a diagnosis of anti-OJ antisynthetase syndrome with severe myositis and interstitial lung disease (ILD) with organizing pneumonia was made.

## 4. Management

Antibiotics were discontinued once infection was ruled out, and nonbacterial thrombotic endocarditis (marantic endocarditis) was diagnosed. A pulmonary embolism was treated with therapeutic anticoagulation. When organizing pneumonia was diagnosed, methylprednisolone 60 mg IV every 6 hours was initiated for 3 days, followed by prednisone 40 mg twice daily. With the progression of myositis and weakness, higher doses of methylprednisolone were resumed (1000 mg daily for 5 days). As oropharyngeal dysphagia developed, a four-day course of intravenous immunoglobulin (IVIG) was started, and prednisone was tapered slowly. Thiopurine methyltransferase (TPMT) enzyme levels were low, so azathioprine was not recommended, and mycophenolate mofetil 500 mg twice daily was initiated. Strength gradually increased, and he underwent physical therapy at an acute rehabilitation center. He continued to improve and was able to return home 5 months after his initial presentation.

## 5. Discussion

Antisynthetase syndrome (ASS) is a rare and complex autoimmune disease, which is a subset of the idiopathic inflammatory myopathies (IIM). IIMs are characterized by varying degrees of skeletal muscle inflammation, predominantly involving the proximal muscles [1]. The annual incidence of IIMs is approximately 1 in 100,000, and they are more common in women [2]. IIMs are categorized into four subgroups: (1) polymyositis (PM), (2) dermatomyositis (DM), (3) sporadic inclusion body myositis, and (4) immune-mediated necrotizing myopathy. ASS is a subset of PM/DM [1]. ASS is characterized by the presence of autoantibodies against aminoacyl-transfer RNA synthetase enzymes, which play a crucial role in protein synthesis. These antibodies include anti-Jo-1, anti-PL-7, anti-PL-12, anti-OJ, anti-EJ, anti-KS, anti-ZO, and anti-tyrosyl antibodies, with the anti-Jo-1 antibody being the most common [3].

Patients with ASS may have variable clinical presentations depending on the specific autoantibody present. Anti-OJ antibodies were detected in our patient. The systemic manifestations of ASS generally include myositis, ILD, arthritis, Raynaud's phenomenon, fissured hyperkeratotic skin changes (mechanic's hands), arthritis, and fever [3]. Formal diagnostic criteria for ASS were introduced by Connors et al. in 2010 and require the presence of an antiaminoacyl tRNA synthetase antibody in addition to one or more of the following clinical features: Raynaud's phenomenon, mechanic's hands, ILD, myositis, arthritis, or unexplained fever [4]. In 2011, Solomon et al. proposed stricter criteria, which require the presence of antiaminoacyl tRNA synthetase antibodies in addition to two major or one major and two minor criteria [5].

Major criteria include the following:

- ILD (not attributable to another cause) and
- Polymyositis or dermatomyositis

Minor criteria include the following:

- Arthritis
- Raynaud's phenomenon
- Mechanic's hands (See Table 1)

In the case we presented, two major Solomon's criteria were met in addition to antisynthetase antibody.

Myositis-specific antibody panel (MSA) is a highly specific test for IIM, which can differentiate between the subtypes of IIM and help in early diagnosis [2]. The specificity of MSA for the clinical diagnosis of IIM ranges from 94.2% to 99.9%. Classic MSA includes antisynthetase antibodies, including anti-Jo-1 and other antisynthetase antibodies, which are detected in both PM and DM, anti-SRP antibodies (antisignal recognition particles), which are detected in PM and necrotizing autoimmune myositis, and anti-Mi-2 antibodies, which are detected in DM. Newer MSA panels also include anti-TIF1*γ*/*α*, anti-MJ/NXP-2, anti-SAE, and anti-MDA5, antibodies mainly detected in DM. (Although anti-MDA5 may be detected in overlap syndromes and anti-HMGCR antibodies in necrotizing autoimmune myositis.) [6, 7].

CT chest can help in diagnosing ILD. Lung biopsy is not essential for confirming the diagnosis, but HRCT patterns have been correlated with pulmonary histologic findings, including (1) cryptogenic organizing pneumonia (COP), characterized by consolidation and linear opacities; (2) nonspecific interstitial pneumonia (NSIP), characterized by ground-glass opacities and irregular linear opacities; (3) usual interstitial pneumonia (UIP), characterized by honeycombing and traction bronchiectasis; and (4) diffuse alveolar damage (DAD), defined by bilateral and extensive consolidation with airspace and ground-glass opacities [8]. MRI of proximal muscle involved can aid in diagnosis of myositis. EMG can differentiate weakness due to inflammatory myopathy from that of neuropathic origin [1].

Myositis activity is typically assessed via elevations in CK and aldolase [9]. Muscle biopsy can aid in diagnosing myositis, with histopathology revealing perifascicular necrosis and perimysial inflammation with macrophages and lymphocytes [1].

Interstitial lung disease, when present, can be severe and determine morbidity and mortality in ASS. The prognosis and response to treatment depend on the severity and type of pulmonary involvement. Poor prognostic factors in the ASS include age >55 years, symptomatic ILD, the UIP pattern, respiratory muscle involvement, and steroid refractory ILD. The presence of these factors may require more aggressive therapy in patients with ILD and anti-Jo‐1 antibodies [8].

Early diagnosis and initiation of treatment are keys to management. Corticosteroids have long been the first-line of treatment for IIMs, although frequently, lung disease recurs with corticosteroid tapering. Additional immunosuppressive agents are added for refractory muscle and/or lung disease and as corticosteroid-sparing agents. Frequently used adjunctive agents include azathioprine, mycophenolate mofetil, tacrolimus, rituximab, and cyclophosphamide.

A multidisciplinary approach with close follow-up and collaboration between rheumatology and pulmonology is essential. Monitoring for progression of ILD with PFTs every 3–6 months and HRCT every 6–12 months is recommended. Patients with progressive ILD refractive to immunosuppressive therapy require referral for lung transplantation [9].

Previous descriptions of ASS-associated coagulopathy presenting with right ventricular thrombus and PE/DVTs have positive antiphospholipid and/or anticardiolipin antibodies [10]. Nonbacterial thrombotic endocarditis (or marantic endocarditis), as noted in our patient, is a rare, noninfectious endocarditis primarily affecting the aortic and mitral valves in hypercoagulable states [11]. The case we presented is the second case of PE and the first case reported of marantic endocarditis in ASS with negative antiphospholipid, lupus, and anticardiolipin antibodies. Further investigation may be warranted to understand the pathophysiology and association of ASS with thromboembolism, especially when the hypercoagulable panel is negative [12].

## 6. Conclusion

Antisynthetase syndrome is a rare autoimmune disease. Diagnosis can be delayed as clinical features are variable and may evolve. ASS can mimic infection and can often go undiagnosed without strong clinical suspicion. ASS can be associated with thromboembolism, which needs further investigation to elucidate the underlying mechanism, especially when the hypercoagulable panel is negative.

## Figures and Tables

**Table 1:** Diagnostic criteria for antisynthetase syndrome.

Connors et al. [4]	Solomon et al. [5]

Required: presence of an antiaminoacyl tRNA synthetase antibody	Required: presence of an antiaminoacyl tRNA synthetase antibody

PLUS one or more of the following clinical features:	PLUS two major or one major and two minor criteria:
(i) Raynaud's phenomenon	Major
(ii) Arthritis	(i) Interstitial lung disease (not attributable to another cause)
(iii) Interstitial lung disease	(ii) Polymyositis or dermatomyositis
(iv) Fever (not attributable to another cause)	Minor
(v) Mechanic's hands (hyperkeratosis with thickened and cracked skin on hands)	(i) Arthritis
	(ii) Raynaud's phenomenon
	(iii) Mechanic's hands

## Data Availability

The clinical case report data used to support the findings of this study are included within the article.

## Abbreviations

ASS: Antisynthetase syndrome
OP: Organizing pneumonia
ILD: Interstitial lung disease
PE: Pulmonary embolism
CK: Creatine kinase
ANA: Antinuclear antibody
PM: Polymyositis
DM: Dermatomyositis
IIM: Idiopathic inflammatory myositis.
